# Rochester Pathological Society

**Published:** 1888-01

**Authors:** Edward B. Angell


### Society Reports.

### *ROCHESTER PATHOLOGICAL SOCIETY.*

*Regular Meeting, October 13, 1887.*

Reported by EDWARD B. ANGELL, M. D.

Dr. B. J. Preston presented a paper on "Retention of Urine in Elderly Men," the cause of which was usually enlargement of the prostate gland. Special emphasis was given to the danger of overlooking vesical distension when there was constant dribbling of urine.

In these cases, relief must ordinarily be secured through catheterization. For this purpose, he recommended that a soft rubber catheter be used first. In case of failure, an old man's silver catheter may be tried. The vertebrated catheter is also very useful in obstinate cases, while, with the aid of an Otis guide, the writer had only failed once, and failure then was due to the presence of a false passage, the result of effort by a former attendant.

If the bladder is greatly over-distended, it is not safe to withdraw its contents suddenly, as fatal collapse has ensued under such circumstanees.

The after-treatment requires washing out of the bladder two or three times a day with a little warm water, to which is added boracic acid or a little carbolic acid. He advised rest in the recumbent posture, the use of anodyne suppositories, frequent cleansings of the bladder, and maintainance of free action of the bowels, with a properly-regulated diet. Avoidance of sexual indulgence, horse-back exercise and sudden colds was particularly enjoined.

In the subsequent discussion of the paper, Dr. E. N. Howard described the method of catheterizing old men at the almshouse. A gum catheter was employed, the wire guide of which was withdrawn about an inch, thus securing steadiness, with sufficient flexibility at the entering end. He suggested that, sometimes, the difficulty may be overcome if the urethra is made taut by stretching the penis upward. The eye of the catheter may be freed from clots by using a syringe with warm water, which, by-the-by, would often assist the introduction of the catheter. He also referred to the importance of keeping diagrams of an old man's urethra from time to time when possible. Cocaine he had found of service in cystitis, using a weak solution as an injection.

Dr. Carpenter cited a case in which he was able to enter the bladder with a linen catheter having an olivary point, after failure with other means.

Dr. Roseboom had relieved one case very materially by the passage of a steel sound.

Dr. Angell suggested electrolysis as worthy of trial, believing that permanent good might be gained thereby, the gland itself reduced in size, and the calibre of the prostatic urethra enlarged.

Dr. Conklin referred to the extreme liability to cystitis in these cases. He had found the injection of a solution of two grains of quinine in half an ounce of warm water very beneficial for the relief of pain and spasm; while spasm was diminished and desire for frequent urination lessened if half an ounce of water was left in the bladder.

Dr. L. H. Smith expressed his preference for a catheter with an olivary point. Often a fold in the mucous membrane interfered with the passage of the catheter. This fold could be easily drawn forward, and the obstruction smoothed out, with the finger in the rectum.

Dr. Weigel referred to the importance of thorough lubrication. By injecting oil into the catheter, much assistance might be gained in obstinate cases.

Dr. Starr had succeeded by passing the catheter as far as possible, and then injecting a solution of cocaine to relieve the congestion and sensitiveness. He was waiting for a patient who would permit him to operate by cutting the gland as in the operation for stone, since the operation in all cases prevents retention of urine.

Dr. Backus said that fifty per cent. of all cases of general paresis have enlarged prostate.

Dr. O'Hare feared enlarged prostate as a forerunner of serious trouble. He had used infusion of barberry and buchu with much benefit.

Dr. W. R. Howard advised the use of mercury to reduce the size of the gland, and the injection of a solution of pepsin to remove blood-clots in the bladder.

In reply to a question as to a means of resort in case of failure by catheterization, Dr. Preston advised supra-pubic aspiration, a method of relief often resorted to of late.

In the report of prevailing diseases, it is worthy of note that twenty-one cases of acute jaundice were recorded, ten of them being children under five years of age.

